# Cytoplasmic HMGB1 promotes the activation of JAK2-STAT3 signaling and PD-L1 expression in breast cancer

**DOI:** 10.1186/s10020-025-01235-0

**Published:** 2025-05-19

**Authors:** Ju-Young Han, Woo Joong Rhee, Jeon-Soo Shin

**Affiliations:** 1 Department of Microbiology, Yonsei University College of Medicine, 50-1 Yonsei-ro Seodaemun-gu, Seoul, 03722 South Korea; 2 Brain Korea 21 FOUR Project for Medical Science, Yonsei University College of Medicine, Seoul, 03722 South Korea; 3 Institute for Immunology and Immunological Diseases, Yonsei University College of Medicine, Seoul, 03722 South Korea

**Keywords:** Breast cancer, HMGB1, JAK2, PD-L1, STAT3

## Abstract

**Background:**

High-mobility group box 1 (HMGB1) plays various roles depending on its subcellular localization. Extracellular HMGB1 interacts with receptors, such as toll-like receptor 4 and receptor for advanced glycation end products (RAGE), promoting cell proliferation, survival, and migration in cancer cells. It also increases the expression of programmed death-ligand 1 (PD-L1) in cancer cells by binding to RAGE. However, the effect of intracellular HMGB1 on the regulation of immune checkpoints such as PD-L1 has not been well characterized. In this study, we aimed to investigate the effects of intracellular HMGB1 on PD-L1 expression in breast cancer cells.

**Methods:**

Human and mouse triple-negative breast cancer cells, MDA-MB-231 and 4T1, along with HMGB1-deficient mouse embryonic fibroblast cells, were cultured. HMGB1 overexpression was achieved using a Myc-tagged plasmid, while siHMGB1 constructs were used for gene silencing. Quantitative reverse-transcriptase PCR and western blot analysis were performed to assess gene and protein expressions. Confocal imaging, immunoprecipitation, and proximity ligation assays were used to investigate HMGB1 localization and Janus kinase 2 (JAK2)–signal transducer and activator of transcription 3 (STAT3) interactions. In vivo experiments were performed using tumor-bearing mice treated with STAT3 and HMGB1 inhibitors. Statistical analyses were performed using Student’s t-tests, one-way analysis of variance, Pearson’s correlation, and Kaplan–Meier survival analysis, with significance set at *p* < 0.05.

**Results:**

In breast cancer cells, HMGB1 translocation from the nucleus to the cytoplasm increased the JAK2-STAT3 interaction and induced STAT3 phosphorylation, leading to increased STAT3 target signaling, including the epithelial-mesenchymal transition (EMT) phenotype and PD-L1 expression. Inhibition of nucleo-cytoplasmic translocation of HMGB1 decreased STAT3 phosphorylation and PD-L1 expression. Furthermore, HMGB1 enhanced breast cancer cell migration, invasion, and EMT, contributing to tumor growth in an in vivo mouse model that were mitigated by the HMGB1-targeted approach.

**Conclusions:**

These findings underscore the critical role of intracellular HMGB1 in modulating PD-L1 expression via the JAK2–STAT3 signaling pathways in breast cancer and suggest that targeting HMGB1 translocation is a promising strategy for breast cancer treatment.

**Graphical Abstract:**

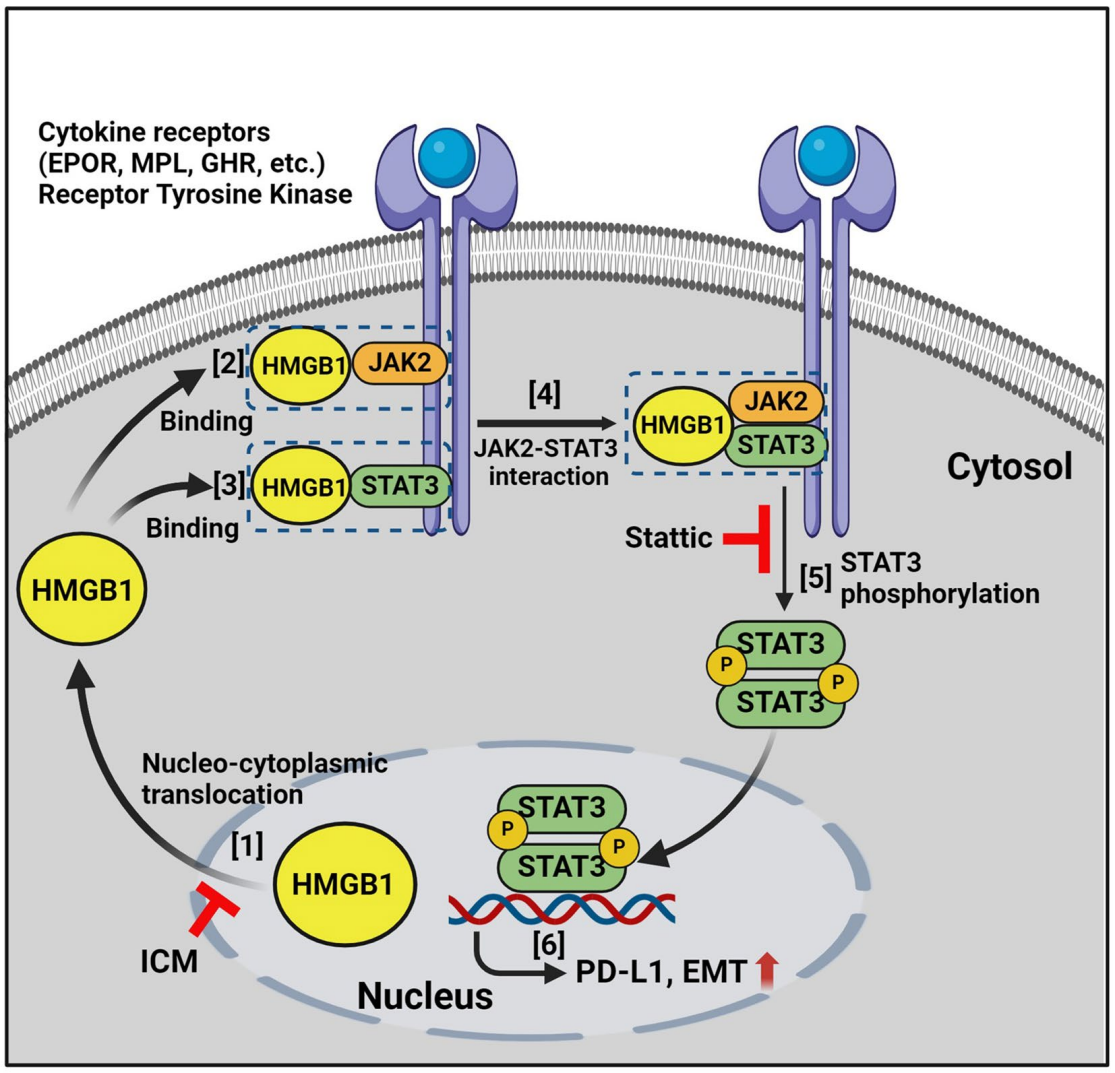

**Supplementary Information:**

The online version contains supplementary material available at 10.1186/s10020-025-01235-0.

## Background

High-mobility group box 1 (HMGB1) is a highly conserved, non-histone nuclear protein that plays diverse roles dependent on its subcellular localization. Within the nucleus, HMGB1 acts as a DNA chaperone, facilitating processes such as DNA replication, V(D)J recombination, DNA repair, and transcription regulation (Bianchi et al. 1989; Goodwin et al. 1973; Javaherian et al. 1978; Lange et al. 2008; Min et al. 2013; Stros 2010; Ueda and Yoshida 2010). In the cytoplasm, HMGB1 influences autophagy, cell survival, and apoptosis (Tang et al. 2010; Zhu et al. 2015). Extracellular HMGB1, released upon cellular stress or damage, acts as a damage-associated molecular pattern (DAMP) binding to receptors, such as the receptor for advanced glycation end products (RAGE) and toll-like receptors (TLRs). This interaction promotes inflammatory responses, tumor growth, and metastasis (Kwak et al. 2015; Lai et al. 2021; Wang and Zhang 2020; Youn et al. 2008). The subcellular localization of HMGB1 is regulated by various post-translational modifications (PTM) such as acetylation (Bonaldi et al. 2003), phosphorylation (Oh et al. 2009; JH Youn and JS Shin, 2006), oxidation (Hoppe et al. 2006; Kwak et al. 2019), ADP-ribosylation (Ditsworth et al. 2007), methylation (Ito et al. 2007), and glycosylation (Kim et al. 2016; Kwak et al. 2020), which facilitates the nucleo-cytoplasmic translocation of HMGB1 and subsequent extracellular secretion (Chen et al. 2022; Gardella et al. 2002).

Breast cancer, one of the most prevalent malignancies affecting women globally (Lei et al. 2021), presents a diverse array of subtypes with varying biological behaviors and prognostic implications (Harbeck et al. 2019). The advancements in early detection and treatment strategies have improved survival rates; however, the prognosis for advanced-stage breast cancer remains challenging. This dichotomy underscores the importance of tailored therapeutic approaches for patients with advanced breast cancer. Especially, the basal-like subtype, often referred to as triple-negative breast cancer (TNBC) owing to its absence of estrogen receptor, progesterone receptor, and human epidermal growth factor receptor 2 (HER2) expression, is of particular concern (Bianchini et al. 2022). TNBC is considerably aggressive, with limited treatment options due to the absence of targeted therapies (Dent et al. 2007), highlighting a significant gap in breast cancer treatment and the urgent need for novel interventions.

In recent years, immuno-oncology has emerged as a promising approach for treating advanced-stage cancers (Waldman et al. 2020). Several clinical trials have highlighted the potential of immune checkpoint inhibitors (ICIs) in patients with TNBC (Liu et al. 2023). Immune checkpoint proteins such as programmed death-ligand 1 (PD-L1), tumor mutational burden (TMB), and tumor-infiltrating lymphocytes (TILs), are used as biomarkers to predict the efficacy of ICIs against cancers, particularly in metastatic TNBC (Geurts and Kok 2023; Muenst et al. 2014; Qureshi et al. 2022). Extracellular HMGB1 upregulates the PD-L1 expression by binding to RAGE, thereby aiding tumor immune evasion (Amornsupak et al. 2022; Wang et al. 2019). However, the role of cytoplasmic HMGB1 on the expression of immune checkpoints remains largely unknown.

In this study, we aimed to investigate the involvement of cytoplasmic HMGB1 in the STAT3 signaling and expression of PD-L1 in breast cancer. Our findings reveal that nucleo-cytoplasmic translocation of HMGB1 increased the JAK2-STAT3 interaction, promoting the phosphorylation of STAT3 and expression of PD-L1. This study will help identify the potential therapeutic strategies targeting the nucleo-cytoplasmic translocation of HMGB1 in breast cancer.

## Materials and methods

### Cell culture, transfection, and reagents

Human TNBC cells (MDA-MB-231; HTB-26; ATCC, VA, USA) and HMGB1-deficient mouse embryonic fibroblast cells (MEF^HMGB1 KO^; HMGBiotech, Milano, Italy) were cultured in Dulbecco’s modified Eagle’s medium (DMEM; Corning Inc., Corning, NY, USA). Mouse TNBC cells (4T1; CRL-2539; ATCC) were cultured in RPMI 1640 medium (Corning Inc.). All media were supplemented with 10% fetal bovine serum (FBS; Life Technologies, Camarillo, CA, USA) and 1% penicillin-streptomycin (15140-122; Gibco, Grand Island, NY, USA) and incubated at 37 °C under 5% CO_2_.

The pCMV plasmid containing Myc-tagged HMGB1 was used for HMGB1 overexpression. Furthermore, expression vectors encoding GFP-tagged HMGB1, HMGB1 NLS1/2E, and Myc-tagged (HMGB1)_2_ were also utilized to facilitate HMGB1 overexpression studies. The siHMGB1 constructs (hs.Ri.HMGB1.13.1 [human]; mm.Ri.Hmgb1.13.1 [mouse]; Integrated DNA Technologies, Coralville, IA, USA) were used for HMGB1 silencing. Plasmid and siRNA transfections were performed using Lipofectamine™ 2000 Transfection Reagent (Invitrogen, Waltham, MA, USA) and Lipofectamine™ RNAiMAX Transfection Reagent (Invitrogen), respectively, according to the manufacturer’s protocol.

Stattic, a STAT3 inhibitor (S7024; Selleckchem, Houston, TX, USA); Inflachromene (ICM), an HMGB1 nucleo-cytoplasmic translocation inhibitor (AOB6225; Gloucester, MA, USA); MK-2206, an AKT inhibitor (S1078; Selleckchem); BMS-345541, a NF-κB p65 phosphorylation inhibitor (S8044; Selleckchem); SCH772984, an ERK1/2 inhibitor (S7101; Selleckchem); dimethyl sulfoxide (D2650; Sigma-Aldrich, St. Louis, MO, USA) were used.

### Quantitative reverse-transcriptase PCR (qRT-PCR) analysis

RNA was extracted using the AccuPrep^®^ Universal RNA Extraction Kit (K-3140; Bioneer Inc., Daejeon, South Korea). A total of 1 µg of RNA was subjected to reverse transcription using RNA to cDNA EcoDry Premix (Oligo dT) (#639543; TAKARA Bio Inc., Kusatsu, Shiga, Japan). qRT-PCR was performed using Power SYBR™ Green PCR Master Mix (4367659; Applied Biosystems, Waltham, MA, USA) on the StepOnePlus™ Real-Time PCR System (Applied Biosystems). The relative expression level of the target genes was quantified by the 2^−ΔΔCt^ method using the Ct value of *GAPDH* as an endogenous control. The primers used in the qRT-PCR analysis included *CD274* forward: CTGCACTTTTA GGAGATTAGATCCTG, *CD274* reverse: TGGGATGACCAATTCAGCTGTA and *HMGB1* forward: TATGGCAAAAGCGGACAAGG and *HMGB1* reverse: CTTCGCAACATCACCAATG GA.

### Western blot analysis

Cells were lysed using RIPA buffer (GenDEPOT, Barker, TX, USA) supplemented with protease (GenDEPOT) and phosphatase inhibitors (Thermo Fisher Scientific, Waltham, MA, USA). Lysates were mixed with protein sample buffer and heated at 98 °C for 5 min. The samples were then subjected to sodium dodecyl sulfate-polyacrylamide gel electrophoresis (SDS-PAGE) and transferred to nitrocellulose membranes. Western blotting was performed using primary antibodies and horseradish peroxidase (HRP)-conjugated secondary antibodies. Antibodies used in the study included anti-HMGB1 (ab18256; Abcam, Cambridge, UK), anti-c-Myc (13-2500; Thermo Fisher Scientific), anti-PD-L1 (17952-1; Proteintech, Hangzhou, China), anti-STAT3 (ab119352; Abcam), anti-p-STAT3 (phosphor Y705) (ab76315; Abcam), anti-AKT (#9272; Cell Signaling Technology, Danvers, MA), anti-p-AKT (#4058; Cell Signaling Technology), anti-ERK1/2 (#9102; Cell Signaling Technology), anti-p-ERK1/2 (#9101; Cell Signaling Technology), anti-p65 (#8242; Cell Signaling Technology), anti-p-p65 (#3033; Cell Signaling Technology), anti-α-Tubulin (#3873; Cell Signaling Technology), anti-Lamin B1 (ab133741; Abcam), and anti-β-actin (ATGA0457; NKMAX, Seongnam, South Korea) antibodies. Protein signals were visualized with an enhanced chemiluminescence substrate (Pierce, Appleton, WI, USA). Images were acquired using an Amersham™ ImageQuant™ 800 (Cytiva, Marlborough, MA, USA).

### Immunoprecipitation (IP) assay

IP assay was performed using SureBeads™ Protein G Magnetic Beads (#1614023; Bio-Rad Laboratories, Hercules, CA, USA). The beads were washed three times with phosphate-buffered saline with 0.5% tween 20 (PBS-T) and incubated with 1 µg of the specific antibody at room temperature (RT) for 1 h. Cell lysates were then added to the antibody-conjugated beads and incubated overnight at 4 °C with rotation. The beads were washed three times with PBS-T, mixed with protein sample buffer, and heated at 98 °C for 10 min. Proteins were separated using SDS-PAGE.

### Nuclear/cytosolic fractionation

Nuclear/cytosolic fractionation was performed with NE-PER™ Nuclear and Cytoplasmic Extraction Reagents (78833; Thermo Scientific) according to the manufacturer’s instructions.

### Confocal imaging

MEF^HMGB1 KO^ were cultured in a Nunc™ Lab-Tek™ II Chamber Slide™ System (154534; Thermo Fisher Scientific), fixed with 4% paraformaldehyde (Biosesang, South Korea), and permeabilized with 0.1% Triton X-100. Intracellular HMGB1 and p-STAT3 were stained with anti-HMGB1 (#MAB1690; R&D Systems, Minneapolis, MN, USA) and anti-p-STAT3 (phosphor Y705) (ab76315; Abcam) antibodies, followed by corresponding fluorochrome-labeled secondary antibodies. After mounting with Fluoromount-G™ Mounting Medium containing DAPI (00-4959-52; Invitrogen™), images were captured using a Zeiss LSM 700 confocal microscope (Carl Zeiss AG, Germany).

### Transwell cell migration and invasion assays

Cells were seeded on Falcon^®^ Permeable Support for 24-well plates (353097; Corning). For the invasion assay, the Transwell inserts were coated with Matrigel (354230; Corning). Then, 700 µL of culture medium supplemented with 10% FBS was added to the bottom of the lower chamber of a 24-well plate. After incubation for 16 h, the cells on the Transwell inserts were fixed with methanol for 20 min. Migrated or invaded cells were then stained with 0.1% crystal violet (548-62-9; Duksan, Ansan, South Korea). Images were captured using a microscope (ECLIPSE TS100; Nikon Corporation, Japan).

### Wound-healing assay

Cells were seeded on Culture-Insert 2 Well in µ-Dish (80206; Ibidi, Gräfelfing, Germany). Once the cells reached confluence and completely covered the wells, the culture inserts were removed, and the medium was replaced with Opti-MEM (31985-070; Gibco). The wound healing capacity was assessed by calculating the percentage of area coverage in each sample at 0 and 24 h.

### Proximity ligation assay (PLA)

The molecular interaction between JAK2 and STAT3 was evaluated using a Duolink^®^ In Situ Red Starter Kit Mouse/Rabbit (DUO92101; Sigma-Aldrich). HMGB1 and p-STAT3 were also observed. MEF^HMGB1 KO^ and MDA-MB-231 cells were transfected with Myc-HMGB1 plasmid and siHMGB1, respectively. Both cells were placed in Nunc™ Lab-Tek™ II Chamber Slide™ System (154534; Thermo Scientific). Cells were fixed with 4% paraformaldehyde at RT for 30 min and stained with anti-JAK2 (ab108596; Abcam), anti-STAT3 (ab119352; Abcam), anti-c-Myc (13-2500; Thermo Fisher Scientific) and anti-p-STAT3 (ab76315; Abcam) antibodies at 4℃ for overnight. After washing, cells were incubated with PLA probe mixture and ligation solution at 37℃. For amplification, a polymerase solution containing fluorescence-labeled oligonucleotides was applied for 90 min at 37℃. The fluorescent spots and images were captured using a Zeiss LSM 700 confocal microscope.

### Mouse experiments

Age- and sex-matched 7-week-old female BALB/c mice were used for the in vivo experiments. All experiments were performed according to the guidelines approved by the Institutional Animal Care and Use Committee of the Yonsei Laboratory Animal Research Center (YLARC, 2022 − 0235). To establish tumors, 4T1 cells were transfected with either the pCMV empty vector or the Myc-HMGB1 plasmid. Subsequently, cells (1 × 10^6^) suspended in 100 µL of phosphate-buffered saline (PBS), were injected into the left flank of each mouse. Once the tumors reached an approximate volume of 150 mm^3^, the mice were treated intraperitoneally with 100 µL of PBS, Stattic (4 mg/kg), or ICM (30 mg/kg) three times per week. To evaluate the effect of HMGB1 expression level, 4T1 cells were transfected with siCtrl or siHMGB1 and injected into the left flank at a concentration of 1 × 10^6^ cells. Tumor sizes were measured three times per week using a caliper, and tumor volumes were calculated using the modified ellipsoidal formula (Euhus et al. 1986): Volume = (Length × Width^2^)/2.

### Immunohistochemical (IHC) staining and histological analysis

For histological analysis, tumor tissues were fixed in 4% paraformaldehyde overnight at 4 °C. Tissue Sect. (4 μm thick) were cut from paraffin-embedded tissue blocks and stained with hematoxylin and eosin (H&E). For antigen retrieval, slides were subjected to microwave irradiation in sodium citrate buffer (pH 6.0). The following primary antibodies were used for IHC analysis: anti-α-SMA (1:300, ab7817; Abcam), anti-CD8a (1:500, ab217344; Abcam), anti-HMGB1 (1:1000, ab18256; Abcam), anti-PD-L1 (1:200, 17952-1; Proteintech), and anti-p-STAT3 (1:400, ab76315; Abcam) antibodies. Signals were detected using an OptiView DAB IHC Detection kit (Ventana Medical Systems, Tucson, AZ, USA). Slide images were captured using a microscope slide scanner Aperio^®^ AT2 (Leica Microsystems, Wetzlar, Germany). The protein expression was measured and quantified using the ImageJ software (National Institutes of Health, Bethesda, MD, USA).

### Public datasets

For survival analysis, the GSE96058 dataset and Kaplan–Meier plots were obtained from KM Plotter (https://kmplot.com/analysis/). To compare HMGB1 expression levels by breast cancer subtype, microarray (MJ van de Vijver et al. 2002), PNAS1732912100, GSE1379, GSE2603, GSE1456. GSE2034, GSE2741, GSE3143, E_TABM_158, GSE4922, GSE7390, GSE6532, GSE5327, E_UCON_1, GSE7849, GSE9893, GSE9195, GSE10510, GSE11264, GSE11264, GSE11121, GSE12093, GSE8757, GSE7378, GSE16391, GSE19615, GSE17907, GSE22219, GSE26971, GSE25055, GSE20685, GSE21653, GSE45255, GSE2109, GSE8193, GSE20462, GSE17705, GSE24450, GSE31448, METABRIC, E_MTAB_365, GSE30682, GSE42568, GSE40115, GSE55338, GSE43358, GSE36295, GSE37751, GSE76274, GSE97177, GSE12276, GSE18864, and GSE86166) and RNA-seq (The cancer genome atlas [TCGA], GSE96058, and GSE81538) datasets were analyzed using bc-GenExMiner v5. 0. (http://bcgenex.ico.unicancer.fr) (Jézéquel et al. 2012).

Gene set enrichment analysis (GSEA) was performed using GSEA software developed by a joint project of the University of California, San Diego, and the Broad Institute (http://www.gsea-msigdb.org/gsea/index.jsp) (Subramanian et al. 2005). GSEA for Breast Invasive Carcinoma (TCGA Firehose Legacy) data was performed using the “STAT3_02” and “GOTZMANN_Epithelial_to_mesenchymal_transition_up” gene sets from the Molecular Signatures Database (MSigDB) version 7.4.

### Statistical analysis

Statistical significance between the two groups was assessed using a two-tailed unpaired Student’s t-test, and a paired t-test was used to analyze paired sample groups. One-way analysis of variance (ANOVA) test with Tukey’s post-hoc correction was used to determine the difference among the three or more groups. Pearson’s correlation analysis was used to determine correlations between the two samples. Kaplan–Meier survival analysis, along with a log-rank test, was used to construct survival curves and evaluate statistical significance. All statistical analyses were performed using SPSS Statistics (version 26.0; IBM, Armonk, NY, USA) and GraphPad Prism (version 10.3.0 for Windows, GraphPad Software, Boston, MA, USA). Statistical significance was set at *p* < 0.05.

## Results

### HMGB1 is upregulated in breast cancer, and its expression is associated with PD-L1

**Fig. 1:**
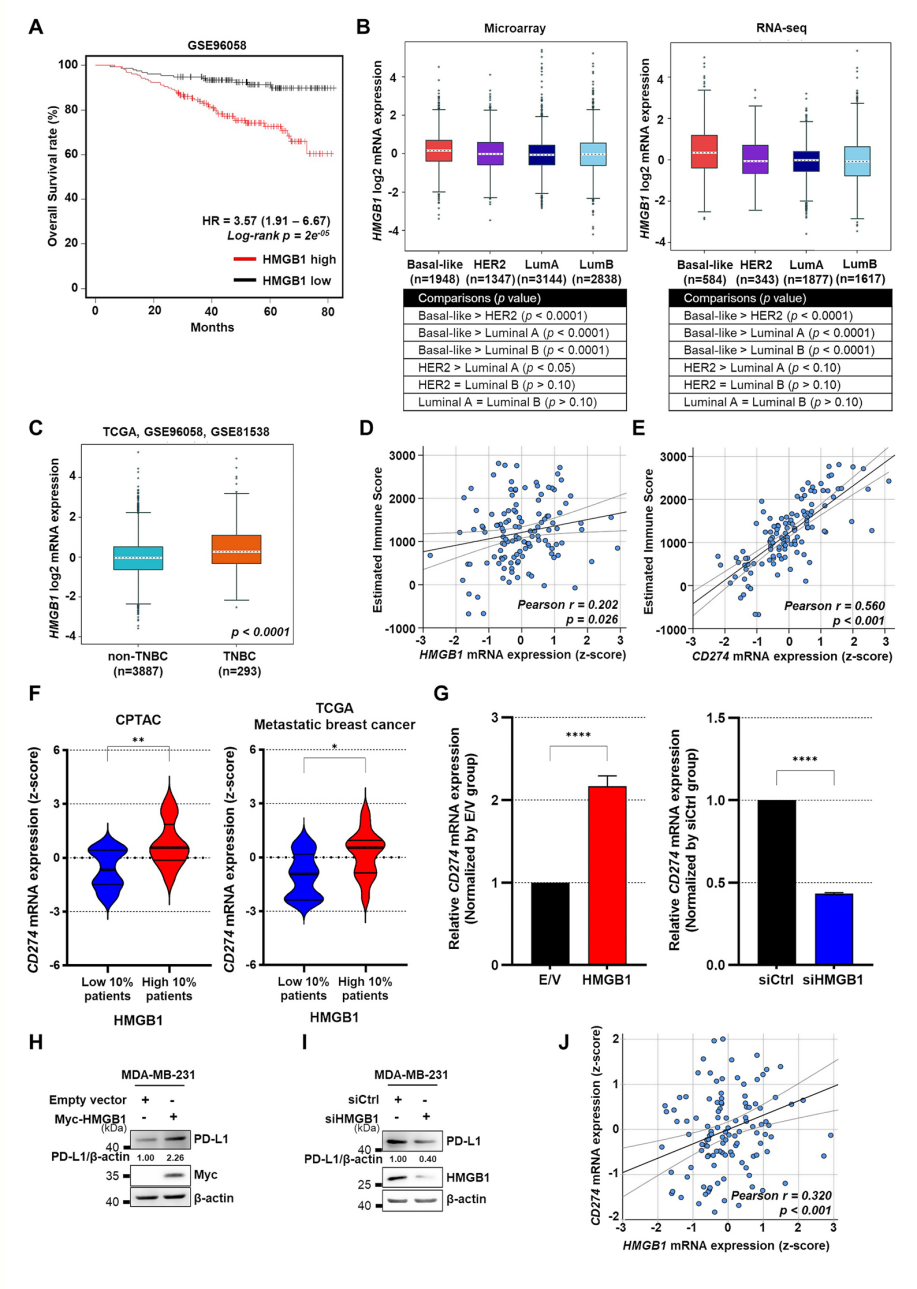
HMGB1 is upregulated in breast cancer and its expression is associated with immune checkpoint genes, including PD-L1. **(A)** Kaplan–Meier plot for overall survival of patients with *HMGB1* high and *HMGB1* low expression based on the GSE96058 dataset using the KM plotter. **(B)***HMGB1* mRNA expression levels among four breast cancer subtypes using the public microarray and RNA-seq datasets obtained from the bc-GenExMiner v5.0. Comparisons between each subtype are shown in the table. **(C)***HMGB1* mRNA expression levels between TNBC and non-TNBC breast cancers from TCGA, GSE96058, and GSE81538 datasets obtained from the bc-GenExMiner v5.0. **(D**,** E)** Pearson’s correlation plot between Estimated Immune Score and *HMGB1***(D)***or CD274***(E)** mRNA expression levels obtained from the CPTAC dataset. **(F)** Violin plots of *CD274* expression in the top and bottom 10% of *HMGB1* expressing patients in the CPTAC and TCGA metastatic breast cancer datasets. **(G)** Relative mRNA expressions of *CD274* between E/V and Myc-HMGB1 groups and siCtrl and siHMGB1 groups; *n* = 3 samples per group; MDA-MB-231 cells were transfected with E/V or Myc-HMGB1 plasmid and incubated for 24 h and those transfected with siCtrl or siHMGB1 were incubated for 48 h to assess the mRNA expression of *CD274* using qRT-PCR. **(H**,** I)** Immunoblots representing the expression of PD-L1 in MDA-MB-231 cells transfected with E/V or Myc-HMGB1 plasmid **(H)** or siCtrl or siHMGB1 **(I)** (*n* = 3 samples per group). **(J)** Pearson’s correlation plot between *HMGB1* and *CD274* mRNA expression levels using the CPTAC dataset. Data are presented as the mean ± SEM. The two-tailed unpaired Student *t*-test was used for statistical analysis. ns, not significant. *p < 0.05 and **** *p* < 0.0001. TNBC, Triple-negative Breast Cancer; TCGA, The Cancer Genome Atlas; CPTAC, Clinical Proteomic Tumor Analysis Consortium; E/V, Empty Vector; HR, hazard ratio

Evaluation of the prognostic significance of *HMGB1* expression by Kaplan–Meier survival analysis using the GSE96058 dataset revealed the association of the increased *HMGB1* expression with poorer overall survival in patients with breast cancer (Fig. 1A). Comparison of the expression of *HMGB1* mRNA in patients with breast cancer with luminal A, luminal B, HER2, and basal-like subtypes using microarray and RNA-seq public datasets revealed significantly higher *HMGB1* mRNA levels in the basal-like subtype than those in other subtypes (Fig. 1B). Furthermore, *HMGB1* expression was also higher in the TNBC subtype than that in non-TNBC subtypes (TCGA, GSE96058, and GSE81538) (Fig. 1C).

Next, we performed a Pearson correlation analysis between the estimated immune score and *HMGB1* mRNA expression in the Clinical Proteomic Tumor Analysis Consortium (CPTAC) dataset to determine the immunologic relevance of *HMGB1* expression in breast cancer. The analysis revealed a positive correlation (Fig. 1D), similar to the correlation observed between the estimated immune score and the well-known immune checkpoint molecule *CD274* (PD-L1) (Fig. 1E). Next, we stratified the patients from the CPTAC and TCGA metastatic breast cancer dataset into *HMGB1*-high and -low groups based on the top and bottom 10% of *HMGB1* mRNA expression, respectively. A comparison between these two groups revealed higher *CD274* mRNA expression in the *HMGB1*-high patient group than that in the *HMGB1*-low patient group (Fig. 1F), suggesting the association between *HMGB1* and *CD274* expression. To validate these findings in vitro, MDA-MB-231 cells were transiently transfected to either overexpress or knock down HMGB1. This in vitro analysis corroborated the positive correlation between *HMGB1* and *CD274* mRNA expression levels observed in the patient dataset (Fig. 1G). Additionally, HMGB1 protein expression was positively correlated with that of PD-L1 (Fig. 1H, I). A direct positive correlation between *HMGB1* and *CD274* expression was further confirmed in the CPTAC dataset (Fig. 1J). Collectively, these data suggest that HMGB1 expression is correlated with the *CD274* level.

### HMGB1 regulates PD-L1 expression through STAT3 signaling

**Fig. 2:**
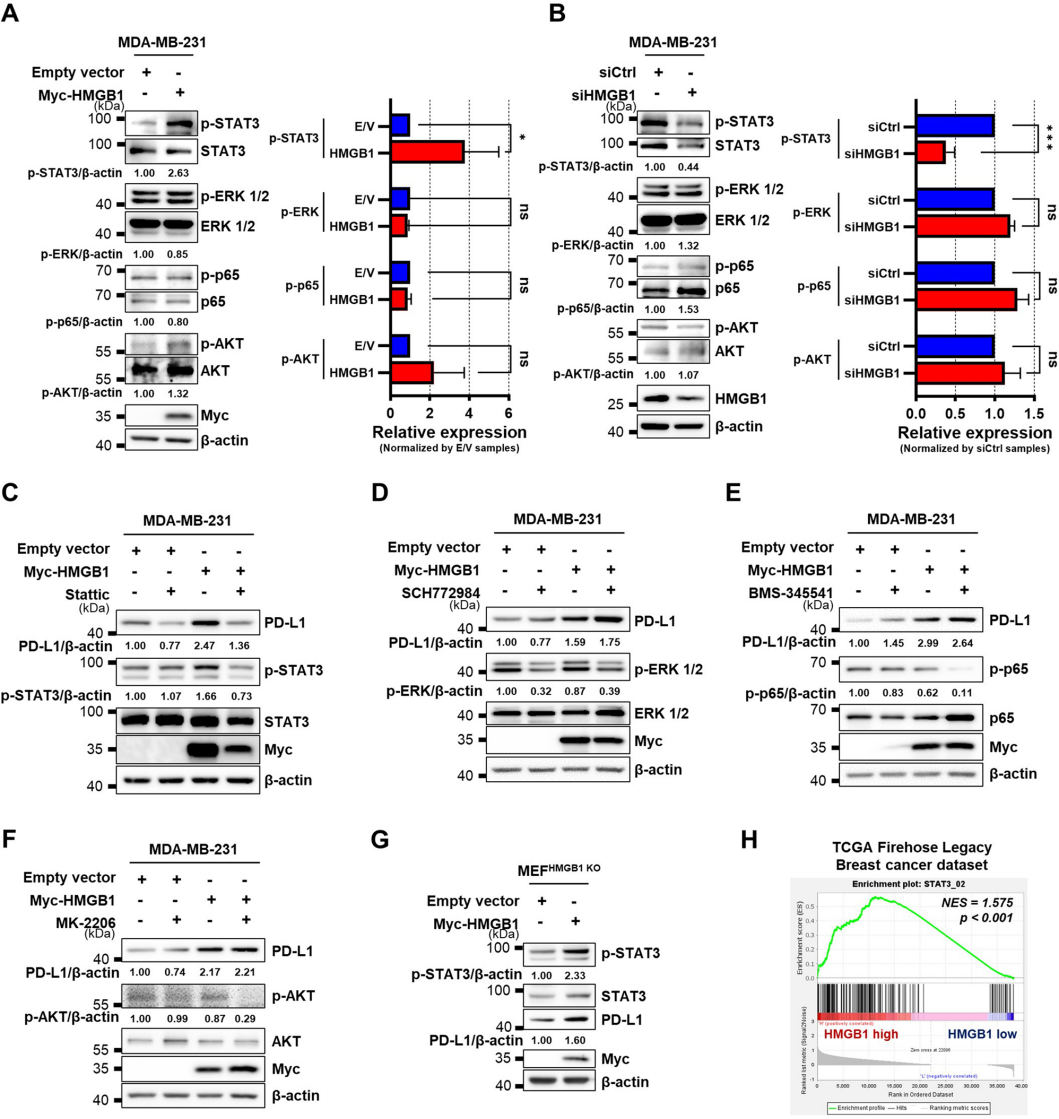
HMGB1 regulates the expression of PD-L1 through the STAT3 signaling. **(A**,** B)** Immunoblots representing the expression of p-STAT3, STAT3, p-ERK1/2, ERK1/2, p-p65, p65, p-AKT, and AKT in MDA-MB-231 cells transfected with empty vector or Myc-HMGB1 plasmid **(A)** or siCtrl or siHMGB1 **(B)**. **(C)** MDA-MB-231 cells were transfected with Myc-HMGB1 for 24 h and treated with 20 µM Stattic for 16 h. **(D)** MDA-MB-231 cells were transfected with Myc-HMGB1 for 48 h and treated with 0.31 µM SCH772984 for 42 h. **(E)** MDA-MB-231 cells were transfected with Myc-HMGB1 for 48 h and treated with 0.62 µM BMS-345541 for 42 h. **(F)** MDA-MB-231 cells were transfected with Myc-HMGB1 for 24 h and treated with 5 µM MK-2206 for 16 h. **(G)** Immunoblots representing the expression of p-STAT3, STAT3, PD-L1 in MEF^HMGB1 KO^ cells transfected with empty vector or Myc-HMGB1. *n* = 3 samples per group for all immunoblot data. **(H)** GSEA enrichment plot for the STAT3 signaling target gene set (STAT3_02) comparing the top 10% and bottom 10% of *HMGB1* expression in the TCGA Firehose Legacy dataset. Data are presented as the mean ± SEM. The two-tailed unpaired Student *t*-test was used for statistical analysis. ns, not significant. *p < 0.05 and *** *p* < 0.001. E/V, Empty Vector; H, Myc-HMGB1; GSEA, Gene Set Enrichment Analysis; NES, normalized enrichment score

Next, we investigated the effects of HMGB1 on PD-L1 expression to elucidate the underlying mechanisms. Oncogenic pathways, including STAT3, ERK1/2, NF-κB, and AKT, have been shown to regulate PD-L1 expression in a variety of cancers (Chen et al. 2016; Fujita et al. 2015; Gowrishankar et al. 2015; Jiang et al. 2013; Lastwika et al. 2016). Assessment of the effects of HMGB1 overexpression or knockdown on the activation of STAT3, ERK1/2, NF-κB p65, and AKT pathways revealed alteration in only p-STAT3 levels in HMGB1-overexpressing or knocked down MDA-MB-231 cells (Fig. 2A, B). HMGB1 could be dimerized by reactive oxygen species, and HMGB1 dimerization could protect DNA in the nucleus and enhance inflammatory response in extracellular stimuli (Kwak et al. 2019, 2021, 2025). To observe whether STAT3 phosphorylation is increased in the presence of the dimer form of HMGB1, the (HMGB1)_2_ plasmid containing two repeats of HMGB1 through an intermediate linker (Kwak et al. 2025) was overexpressed in MDA-MB-231 cells. p-STAT3 levels were increased. These results suggest that HMGB1 dimerization enhances STAT3 phosphorylation (Supplementary Fig. S1). To confirm these findings, we treated HMGB1-overexpressing MDA-MB-231 cells with inhibitors of STAT3 (Stattic), ERK1/2 (SCH772984), NF-κB p65 (BMS-345541), and AKT (MK-2206); among these, PD-L1 expression was reduced only in Stattic-treated cells (Fig. 2C–F). Furthermore, HMGB1 overexpression in HMGB1-deficient MEF cells (MEF^HMGB1 KO^) increased STAT3 phosphorylation and PD-L1 expression (Fig. 2G). Similar to MDA-MB-231 cells, HMGB1-overexpressing 4T1 cells also showed increased STAT3 phosphorylation and PD-L1 expression, whereas the knockdown of HMGB1 showed the opposite effect (Supplementary Fig. S2A, B). Consistently, STAT3 phosphorylation and PD-L1 expression were reduced in Stattic-treated HMGB1-transfected 4T1 cells (Supplementary Fig. S2C). In GSEA using Breast Invasive Carcinoma data (TCGA, Firehose Legacy), the ‘STAT3_02 gene set’ representing *STAT3* target genes was enriched in patients with high HMGB1 expression compared to those with low HMGB1 expression (Fig. 2H). These findings demonstrate that HMGB1 overexpression leads to the phosphorylation of STAT3 and promotes PD-L1 expression.

### HMGB1 binds to both JAK2 and STAT3 and enhances the binding of JAK2 and STAT3

**Fig. 3:**
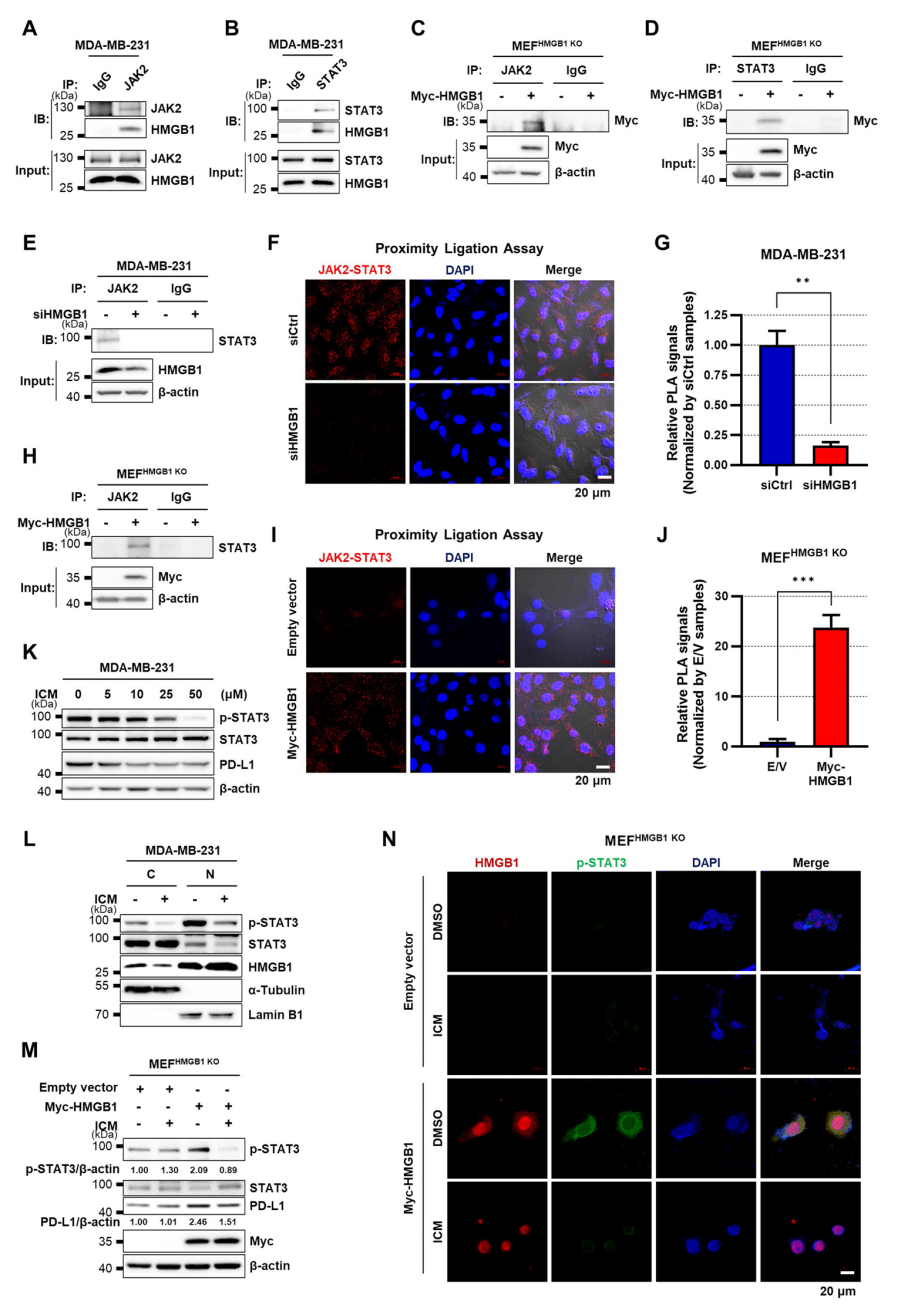
HMGB1 binds to both JAK2 and STAT3 and enhances the binding of JAK2 and STAT3. **(A)** MDA-MB-231 WCLs were immunoprecipitated against IgG or JAK2 and immunoblotted with anti-JAK2 and anti-HMGB1 antibodies. **(B)** MDA-MB-231 WCLs were immunoprecipitated against IgG or STAT3 and immunoblotted with anti-STAT3 and anti-HMGB1 antibodies. **(C)** MEF^HMGB1 KO^ cells were transfected with E/V or Myc-HMGB1 plasmids and incubated for 24 h. WCLs were immunoprecipitated against IgG or JAK2 and immunoblotted with anti-Myc antibody. **(D)** MEF^HMGB1 KO^ cells were transfected with E/V or Myc-HMGB1 plasmids and incubated for 24 h. WCLs were immunoprecipitated against IgG or STAT3 and immunoblotted with anti-Myc antibody. **(E)** MDA-MB-231 cells were transfected with siCtrl or siHMGB1 and incubated for 48 h. WCLs were immunoprecipitated against IgG or JAK2 and immunoblotted with anti-STAT3 antibody. **(F**,** G)** The interaction between JAK2 and STAT3 assessed using PLA (**F**) and quantification of the relative PLA signals **(G)**. MDA-MB-231 cells were transfected with siCtrl or siHMGB1 and incubated for 48 h. **(H)** MEF^HMGB1 KO^ cells were transfected with E/V or Myc-HMGB1 plasmids and incubated for 24 h. WCLs were immunoprecipitated against IgG or JAK2 and immunoblotted with anti-STAT3 antibody. **(I**,** J)** The interaction between JAK2 and STAT3 assessed using PLA (**I**) and quantification of the relative PLA signals **(J)**. MEF^HMGB1 KO^ cells were transfected with E/V or Myc-HMGB1 plasmids and incubated for 24 h. **(K)** Representative immunoblots of p-STAT3, STAT3, and PD-L1 expression in MDA-MB-231 cells treated with 0, 5, 10, 25, and 50 µM ICM for 6 h. **(L)** MDA-MB-231 cells were treated with 25 µM ICM for 6 h. WCLs were separated into cytosolic and nuclear fractions and α-Tubulin and Lamin B1 were used as cytosolic and nucleus markers, respectively. **(M)** MEF^HMGB1 KO^ cells were transfected with E/V or Myc-HMGB1 plasmids and incubated for 48 h, followed by treatment with 25 µM ICM for 6 h. **(N)** The intracellular localization of HMGB1 (red) and p-STAT3 (green) visualized using confocal imaging. For all data, *n* = 3 samples per group. The two-tailed unpaired Student *t*-test was used for statistical analysis. ** *p* < 0.01 and *** *p* < 0.001. WCL, whole cell lysate; IP, immunoprecipitation; IB, immunoblot; E/V, empty vector; ICM, inflachromene; PLA, proximity ligation assay

JAK2 plays a pivotal role in the regulation of STAT3 activation and functions as an upstream molecule of STAT3 (Hu et al. 2021). Next, to understand the mechanism through which HMGB1 activates STAT3, we performed an IP assay to assess the binding of HMGB1 to JAK2 and STAT3. Interestingly, endogenous HMGB1 binds to JAK2 and STAT3 in MDA-MB-231 cells without external stimuli (Fig. 3A, B). Furthermore, overexpression of exogenous HMGB1 also revealed the binding to JAK2 and STAT3 in MEF^HMGB1 KO^ cells (Fig. 3C, D). Knocking down HMGB1 expression using siHMGB1 in MDA-MB-231 cells resulted in reduced JAK2 and STAT3 binding, as evidenced by IP and PLA (Fig. 3E–G). Conversely, HMGB1 overexpression in MEF^HMGB1 KO^ cells augmented the interaction between JAK2 and STAT3 (Fig. 3H–J). Collectively, HMGB1 binds to both JAK2 and STAT3, facilitating their interaction, highlighting the critical role of HMGB1 in modulating the JAK2-STAT3 axis. HMGB1 consists of two DNA binding domains, the A and B boxes. The A and B boxes were separately overexpressed in MDA-MB-231 cells to determine which domain binds to STAT3. Immunoprecipitation (IP) assay suggests that STAT3 binds to both the A box and the B box (Supplementary Fig. S3A). To determine whether both p-STAT3 and HMGB1 remain co-localized in the nucleus upon HMGB1 overexpression, we performed a PLA assay to observe the binding. As shown in Supplementary Fig. S3B, HMGB1 and p-STAT3 colocalized in the nuclei in addition to the cytoplasm.

Under resting status, HMGB1 is predominantly located in the nucleus, but upon stimulation, it translocates to the cytoplasm and is subsequently secreted (Chen et al. 2022; Kwak et al. 2019, 2020). In contrast, STAT3 is generally localized in the cytoplasm and translocates to the nucleus upon phosphorylation by JAK2 to regulate transcriptional activities (Yu et al. 2009). We assessed the effects of treatment with ICM, an inhibitor of HMGB1 nucleo-cytoplasmic translocation (Kim et al. 2018; Lee et al. 2014), on STAT3 phosphorylation and PD-L1 expression. In MDA-MB-231 cells, ICM treatment decreased the levels of p-STAT3 and PD-L1 in a dose-dependent manner (Fig. 3K). Furthermore, nuclear and cytoplasmic fractionation revealed that ICM treatment decreased cytoplasmic HMGB1 level and both cytoplasmic and nuclear p-STAT3 levels (Fig. 3L). ICM also reduced exogenous HMGB1-induced STAT3 phosphorylation and PD-L1 expression in MEF^HMGB1 KO^ cells (Fig. 3M). In 4T1 cells, STAT3 phosphorylation and PD-L1 expression, which were increased by HMGB1 overexpression, were reduced by ICM treatment (Supplementary Fig. S3C). Confocal imaging also showed that ICM inhibited the nucleo-cytoplasmic translocation of HMGB1 and decreased STAT3 phosphorylation in the MEF^HMGB1 KO^ cells with HMGB1 overexpression (Fig. 3N). Next, we used the HMGB1 NLS1/2E plasmid, which contains the substitution of serine residues with glutamic acid within the nuclear localization sequence (NLS) to mimic phosphorylation, resulting in the cytoplasmic location (JH Youn and JS Shin, 2006). Overexpression of HMGB1 NLS1/2E resulted in significantly increased p-STAT3 levels compared to empty vector (Supplementary Fig. S3D).

### HMGB1 promotes MDA-MB-231 migration and invasion by enhancing the STAT3 signaling pathway

**Fig. 4:**
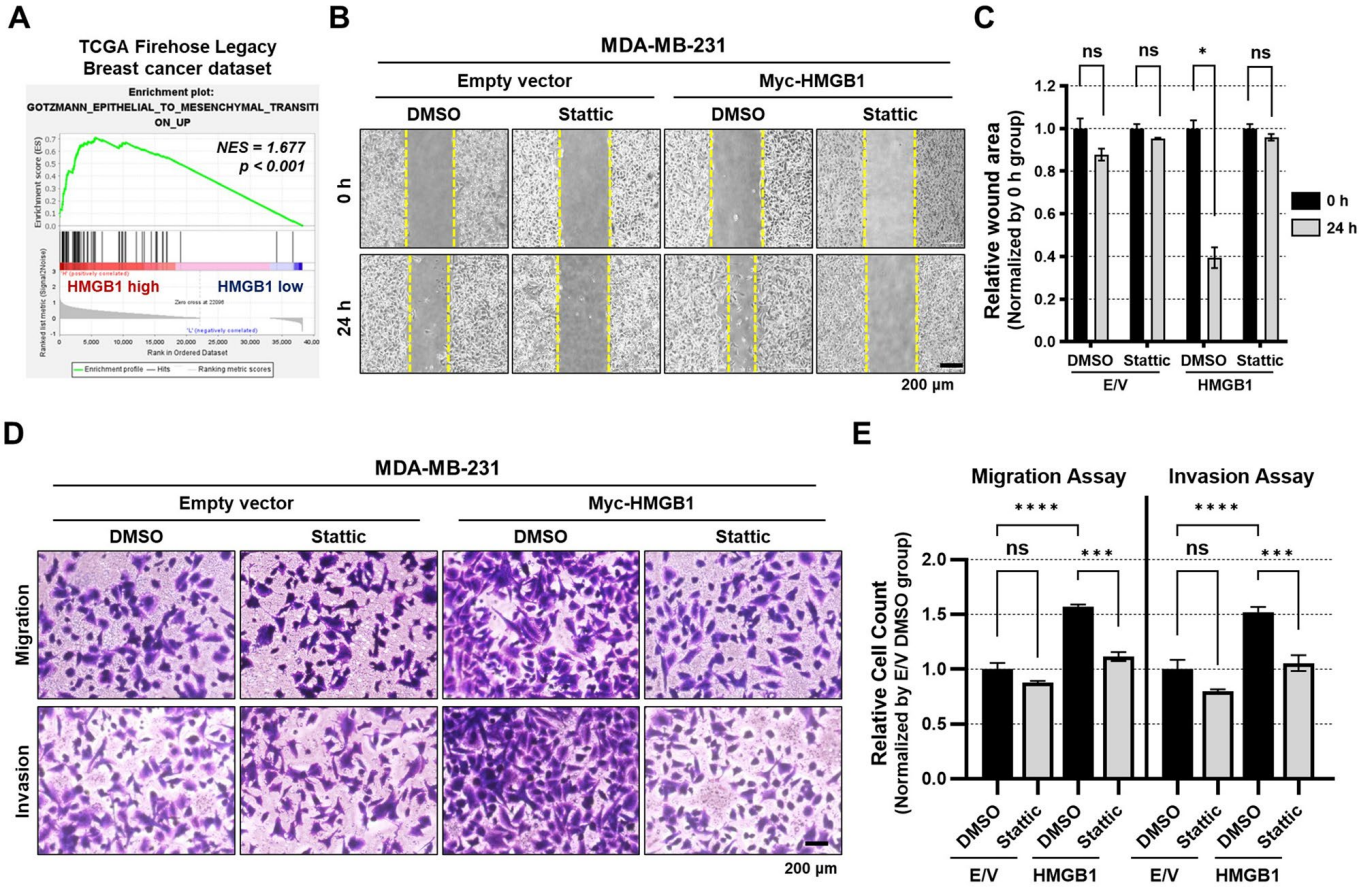
HMGB1 promotes the migration and invasion of breast cancer cells by enhancing the STAT3 signaling pathway. **(A)** Enrichment plot for the ′GOTMANN_EPITHELIAL_TO_MESENCHYMAL_TRANSITION_UP′ gene set comparing patients with the top and bottom 10% of *HMGB1* expression in the TCGA Firehose Legacy dataset. **(B**,** C)** Wound-healing assays (**B**) and quantification of relative wound area **(C).** MDA-MB-231 cells were transfected with E/V or Myc-HMGB1 and incubated for 24 h. Wound-healing assays were performed after treatment with Stattic for 16 h and the relative wound area was quantified; *n* = 3 samples per group; Data are presented as the mean ± SEM; *p < 0.05 obtained using a two-tailed unpaired Student *t*-test. **(D**,** E)** Transwell migration and invasion assays (**D**) and quantification of relative invasion and migration **(E).** MDA-MB-231 cells were transfected with E/V or Myc-HMGB1 and incubated for 24 h. Transwell migration and invasion assays were performed after treatment with Stattic for 16 h and relative invasion and migration were quantified; *n* = 3 samples per group; Data are presented as the mean ± SEM; ns, not significant, *** *p* < 0.001, and **** *p* < 0.0001 obtained using a one-way ANOVA test. NES, normalized enrichment score; TCGA, the cancer genome atlas; E/V, empty vector

The STAT3 signaling pathway enhances the EMT phenotypes as well as the expression of PD-L1, which is important for cancer metastasis and resistance (Sadrkhanloo et al. 2022). We performed GSEA to assess whether HMGB1 expression affects EMT phenotypes through STAT3 signaling. The analysis revealed that EMT-related genes were significantly enriched in patients with high HMGB1 expression (Fig. 4A). In MDA-MB-231 cells, an in vitro scratch assay demonstrated that HMGB1 overexpression increased cell migration, which was subsequently reduced by Stattic treatment (Fig. 4B, C). In addition, the transwell migration and invasion assays showed that HMGB1 overexpression increased cell migration and invasion capabilities, which were reduced by Stattic treatment (Fig. 4D, E). These findings provide evidence that HMGB1 not only modulates PD-L1 expression but also EMT phenotypes through the STAT3 signaling pathway.

### Cytoplasmic HMGB1 promotes mouse tumor growth through STAT3 signaling

**Fig. 5:**
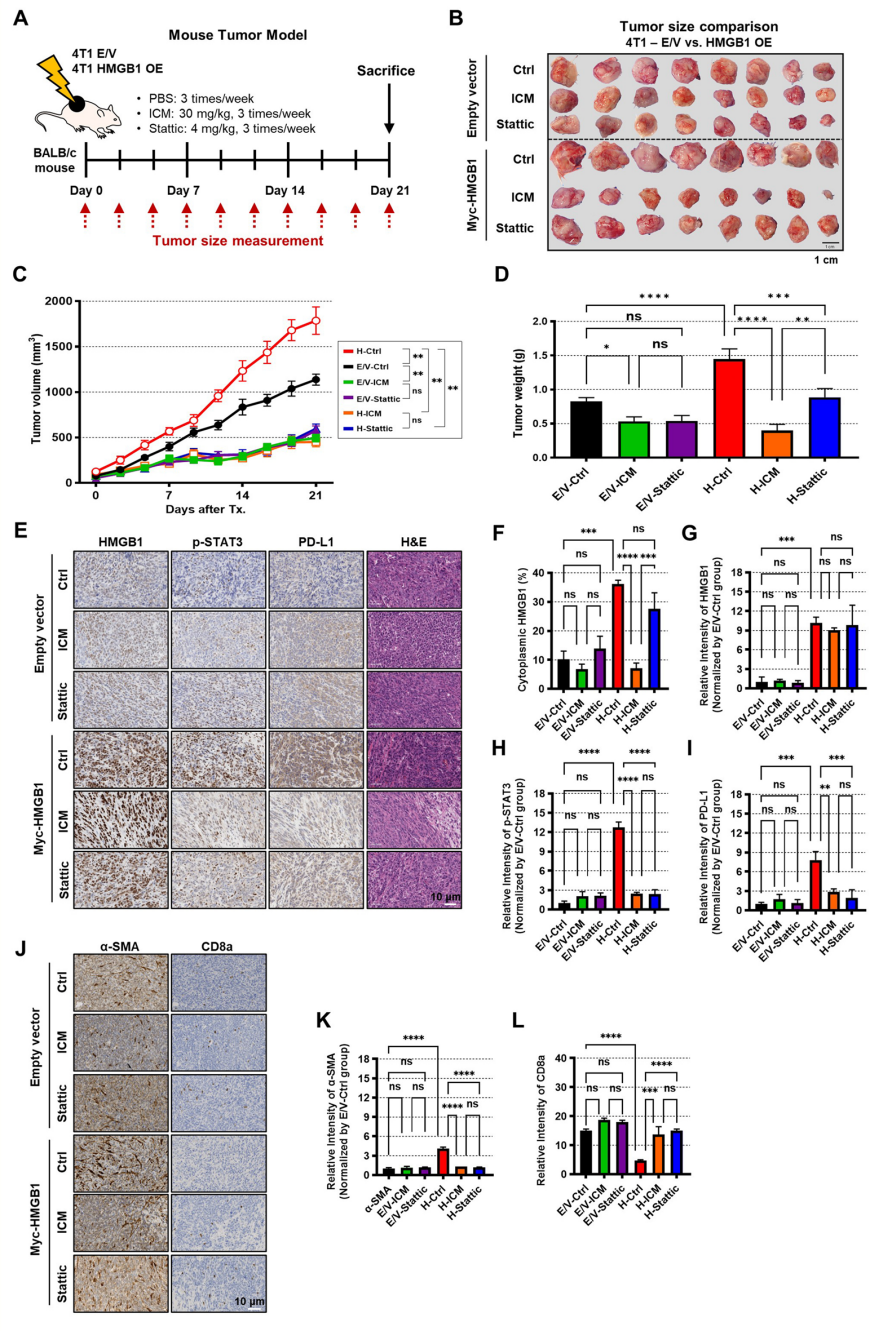
Cytoplasmic HMGB1 promotes mouse tumor growth through STAT3 signaling. **(A)** Experimental scheme of the mouse study. 4T1 cells were overexpressed with E/V (4T1 E/V) or Myc-HMGB1 plasmids (4T1 OE) and implanted to BALB/c mouse. **(B–D)** Tumor images **(B)**, volumes **(C)** and weights **(D)** from each group; *n* = 8 mice per group. **(E**,** J)** Representative IHC images stained with HMGB1, p-STAT3, PD-L1, α-SMA, CD8a and H&E. **(F–I)** Percentage of the cytoplasmic HMGB1 **(F)** and the relative intensities of HMGB1 **(G)**, p-STAT3 **(H)**, and PD-L1 **(I). (K**,** L)** Relative intensity of α-SMA **(K)** and CD8a **(L);***n* = 3 samples per group. Data are presented as the mean ± SEM. ns, not significant, *p < 0.05, ** *p* < 0.01, *** *p* < 0.001, and **** *p* < 0.0001 obtained using one-way ANOVA tests among different groups. E/V, empty vector; OE, overexpression; Tx, Treatment

The regulation of STAT3 activation and PD-L1 expression by HMGB1 was further validated in a mouse model (Fig. 5A). As shown in Fig. 5B, the tumor volume and weight appeared to increase in HMGB1-overexpressing 4T1 cells (H-Ctrl) compared to that in empty vector-transfected (E/V-Ctrl) cells, while treatment with ICM (H-ICM) or Stattic (H-Stattic) reduced the increases. The estimation plot for the paired t-test analysis consistently revealed that the tumor volumes in the H-Ctrl group were significantly larger than those in the E/V-Ctrl group. Tumor volume was also reduced significantly in the E/V-ICM and E/V-Stattic groups compared to that in the E/V-Ctrl group. However, the reductions in tumor volume and weight were more pronounced in H-ICM and H-Stattic samples compared to those in H-Ctrl group (Fig. 5C, D; Supplementary Fig. S4A–G). IHC staining analysis also showed increased intensities of HMGB1, p-STAT3, and PD-L1 in H-Ctrl samples compared to that in the E/V-Ctrl samples, which were reduced by ICM and Stattic treatment (Fig. 5E–I). Specifically, ICM treatment decreased the percentage of cytoplasmic HMGB1 (Fig. 5F), leading to a decrease in STAT3 phosphorylation and PD-L1 expression (Fig. 5H, I). To investigate the potential effects of increased HMGB1 on epithelial-mesenchymal transition (EMT) and T cells, IHC staining was conducted. α-Smooth muscle actin (α-SMA), the EMT marker, was significantly increased in HMGB1-overexpressed tumor tissues and was reduced by ICM and Stattic treatments. And the number of CD8^+^ T cells was increased (Fig. 5J-L).

**Fig. 6:**
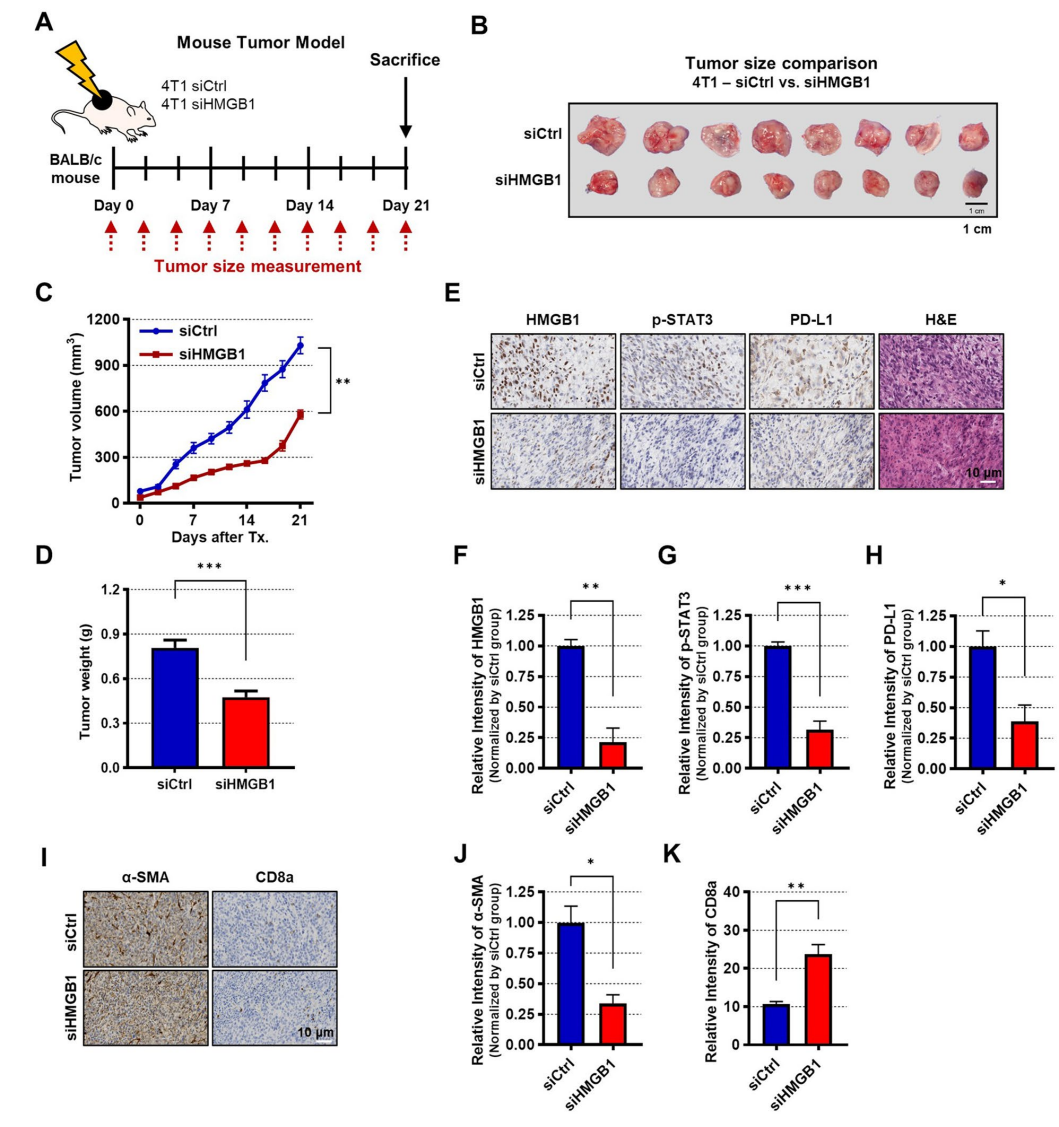
Silencing HMGB1 reduces mouse tumor growth through STAT3 signaling. **(A)** Experimental scheme of the mouse study. 4T1 cells were transfected with siCtrl (4T1 siCtrl) or siHMGB1 (4T1 siHMGB1) and implanted to BALB/c mouse. **(B–D)** Tumor images **(B)**, volumes **(C)** and weights **(D)** from each group; *n* = 8 mice per group. **(E**,** I)** Representative IHC images stained with HMGB1, p-STAT3, PD-L1, H&E **(E)**, α-SMA, and CD8a **(I)**. **(F-H**,** J-K)** Relative intensities of HMGB1 **(F)**, p-STAT3 **(G)**, PD-L1 **(H)**, α-SMA **(J)**, and CD8a **(K)**; *n* = 3 samples per group. Data are presented as the mean ± SEM. *p < 0.05, ** *p* < 0.01, and *** *p* < 0.001 assessed using the two-tailed unpaired Student *t*-test was used for statistical analysis

To assess the effect of HMGB1 knockdown on tumor growth in mice, 4T1 siHMGB1 and 4T1 siCtrl cells were used (Fig. 6A). Tumor sizes in mice injected with siHMGB1 and siCtrl are shown in Fig. 6B. The tumor volume and weight in the siHMGB1 group were significantly smaller than those in the siCtrl group (Fig. 6C, D). The estimation plot showed consistent results (Supplementary Fig. S4H). Furthermore, IHC staining revealed lower intensities of p-STAT3 and PD-L1 in the siHMGB1 group than those in the siCtrl group (Fig. 6E–H). Similarily siHMGB1-treated 4T1 mouse tumor IHC showed the low level of α-SMA and increased level of CD8^+^ T cells (Fig. 6I-K).

**Fig. 7:**
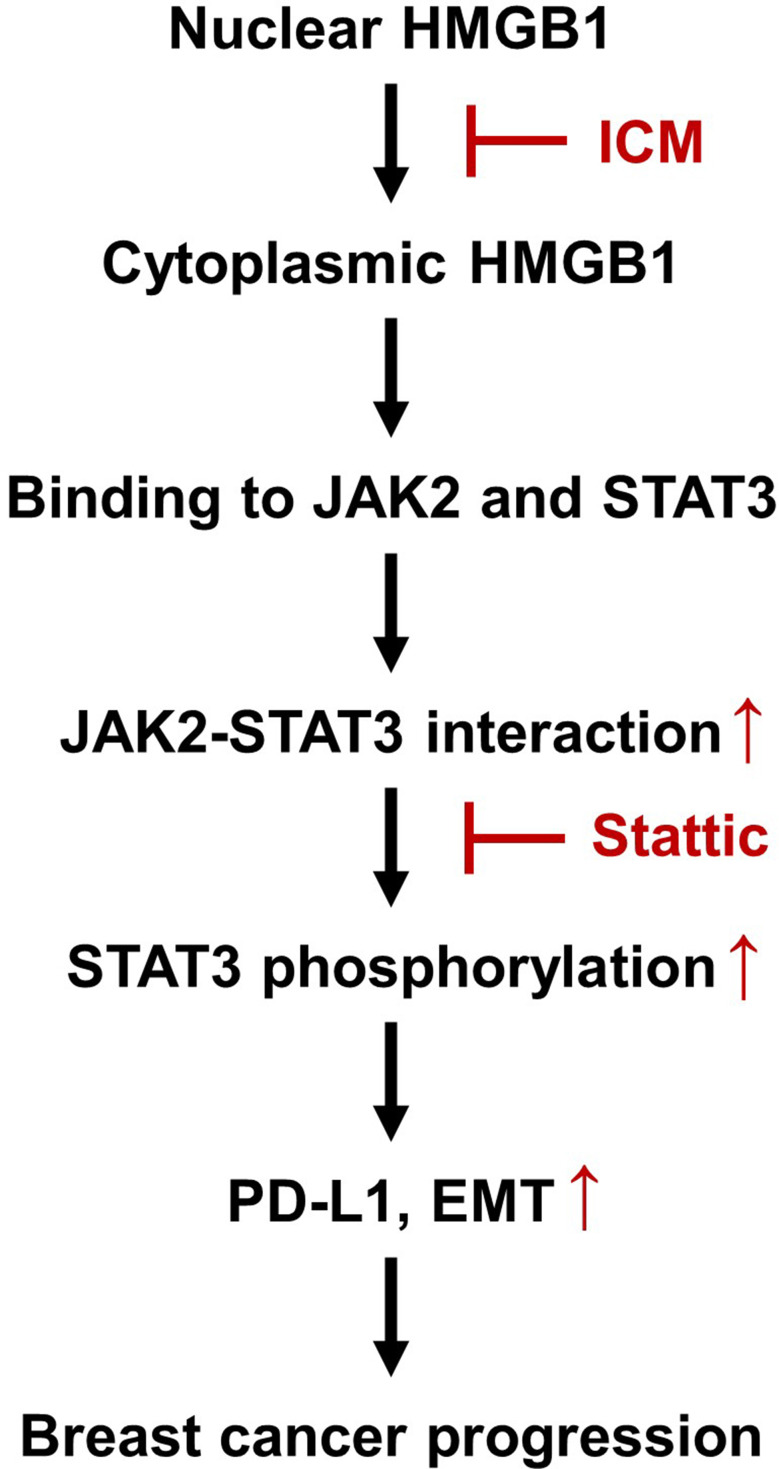
Schematic summary of the proposed mechanism. Cytoplasmic HMGB1 binds to JAK2 and STAT3, increasing STAT3 phosphorylation by enhancing the JAK2-STAT3 interaction. Phosphorylated STAT3 drives EMT by upregulating PD-L1 expression, leading to breast cancer progression. ICM and Stattic, which block the nucleocytoplasmic translocation of HMGB1 and inhibit STAT3 phosphorylation, respectively, resulting in the inhibition of breast cancer progression

Together, these findings demonstrate that cytoplasmic HMGB1 binds to JAK2 and STAT3 and enhances their interaction, leading to increased STAT3 phosphorylation, thereby increasing PD-L1 expression and EMT phenotype (Fig. 7). These findings underscore the pivotal role of HMGB1 in tumor metastasis and progression, highlighting its potential as a crucial target for enhancing the efficacy of immunotherapy.

## Discussion

In this study, we elucidated the role of cytoplasmic HMGB1 in regulating PD-L1 expression through the JAK2-STAT3 signaling pathway. Our findings demonstrate a significant positive correlation between HMGB1 and PD-L1 expression in breast cancer patient data, cellular models, and animal experiments. We showed that HMGB1 enhances PD-L1 expression by promoting the JAK2-STAT3 signaling, one of the upstream regulators of PD-L1. Furthermore, cytoplasmic HMGB1-mediated STAT3 phosphorylation induced STAT3 target genes, including EMT phenotypes as well as PD-L1 expression, and contributed to tumor progression in mice. These findings highlight that the interaction of cytoplasmic HMGB1 with JAK2 and STAT3 is crucial for increasing PD-L1 expression and promoting EMT phenotypes.

In the tumor microenvironment, extracellular HMGB1 increases various inflammatory cytokines and chemokines and promotes tumor growth, angiogenesis, and metastasis through TLR4 and RAGE (Hubert et al. 2021; Idoudi et al. 2023). Moreover, extracellular HMGB1 can increase PD-L1 expression in cancer cells through the activation of the PI3K-AKT pathway after binding to RAGE, thus boosting migration and invasion (Amornsupak et al. 2022). However, to our knowledge, no studies have shown the effect of cytoplasmic HMGB1 on PD-L1 expression. Here, we demonstrate that HMGB1 expression is correlated with PD-L1 expression in both human (MDA-MB-231) and mouse (4T1) cell lines, as well as in MEF^HMGB1 KO^ cells. Furthermore, tumor formation in 4T1 cells supports that the cytoplasmic HMGB1 expression is related to p-STAT3 and PD-L1 expression. In patients with breast cancer, high *HMGB1* levels showed a significantly poorer overall survival rate than low *HMGB1* levels. These results indicate the important role of the cytoplasmic HMGB1 in the regulation of PD-L1 expression and tumor progression.

Our study also highlights that STAT3 phosphorylation is influenced by HMGB1 expression even in the absence of external stimuli. Previous studies have shown that HMGB1 translocated to the cytoplasm due to PTMs of nuclear HMGB1, such as oxidation, phosphorylation, and acetylation (Bonaldi et al. 2003; Hoppe et al. 2006; Kwak et al. 2019; Oh et al. 2009). In addition, many cancer cells, including breast cancer cells of MDA-MB-231, are capable of cytoplasmic translocation of HMGB1 by the PTMs in the absence of external stimuli (Hubert et al. 2021; Kang et al. 2009). In our experiments, MDA-MB-231 and 4T1 cells exhibited basal levels of cytoplasmic HMGB1, and inhibition of its cytoplasmic translocation using ICM inhibited STAT3 phosphorylation and then PD-L1 expression. The JAK2-STAT3 pathway is frequently constitutively activated in various cancers, including breast (K Banerjee and H Resat 2016), gastric (Kanda et al. 2004), prostate (Ni et al. 2000), and pancreatic cancer (Lian et al. 2004), leading to tumor progression, survival, treatment resistance, metastasis, and immune evasion (Hu et al. 2024). Our findings showed that ICM treatment inhibited the phosphorylation of constitutively activated STAT3, suggesting that targeting the nucleo-cytoplasmic translocation of HMGB1 may offer a potential therapeutic strategy against cancers with constitutive STAT3 activation. Furthermore, using PLA and IP assays, we confirmed the binding of HMGB1 to both JAK2 and STAT3 and the increased interaction between JAK2 and STAT3 following HMGB1 overexpression. Nevertheless, future studies are required to investigate the formation of these trimer complexes and explore the mechanism of STAT3 release from HMGB1.

PD-L1 is well-known for its role in tumor immune evasion by binding to the programmed cell death protein 1 receptor on T cells, leading to the inhibition of cytotoxic T cell function and promoting immune tolerance (DS Chen and I Mellman 2013). Beyond immune evasion, PD-L1 has intrinsic roles in cancer cells, including promoting tumor proliferation by activating the AKT/mTOR signaling pathway (Clark et al. 2017), contributing to cancer stemness (Almozyan et al. 2017; Gao et al. 2019), and facilitating metastasis by promoting EMT phenotypes (Chen et al. 2021; Jeong et al. 2024). Our findings demonstrated that HMGB1 overexpression led to increased PD-L1 expression and larger tumor size compared to controls, whereas HMGB1 knockdown resulted in decreased PD-L1 levels and smaller tumors. These observations suggest that future studies are highly needed to clarify whether the effect of PD-L1, regulated by cytoplasmic HMGB1, on tumor size and growth was an immune evasion function or intrinsic tumor property.

In conclusion, the present study reveals a novel mechanism by which cytoplasmic HMGB1 regulates PD-L1 expression through the JAK2-STAT3 signaling pathway. It demonstrates that HMGB1 binds directly to both JAK2 and STAT3, promoting STAT3 phosphorylation and subsequently increasing PD-L1 expression. Inhibiting the nucleo-cytoplasmic translocation of HMGB1, particularly through ICM treatment, reduces STAT3 activation and PD-L1 expression, leading to decreased tumor growth in vivo. Our findings suggest that the HMGB1-targeted approach, which specifically inhibits the nucleo-cytoplasmic translocation of HMGB1, could provide an innovative therapeutic avenue in cancer treatment.

## Electronic supplementary material

Below is the link to the electronic supplementary material.

Supplementary Material 1

## Data Availability

No datasets were generated or analysed during the current study.

## Abbreviations

Ctrl: Control
E/V: Empty vector
EMT: Epithelial to mesenchymal transition
GSEA: Gene set enrichment analysis
H&E: Hematoxylin and eosin
HER2: Human epidermal growth factor receptor 2
HMGB1: High-mobility group box 1
ICI: Immune checkpoint inhibitor
ICM: Inflachromene
IHC: Immunohistochemistry
IP: Immunoprecipitation
JAK2: Janus kinase 2
MEF^HMGB1 KO^: HMGB1-deficient MEF
PD-L1: Programmed death-ligand 1
PLA: Proximity ligation assay
PTM: Post-translational modification
RAGE: Receptor for advanced glycation end products
STAT3: Signal transducer and activator of transcription 3
TCGA: The cancer genome atlas
TIL: Tumor infiltrating lymphocyte
TLR: Toll-like receptor
TMB: Tumor mutation burden
TNBC: Triple-negative breast cancer
