# Supplementary material for: Cytoplasmic HMGB1 promotes the activation of JAK2-STAT3 signaling and PD-L1 expression in breast cancer
Source: Mol Med. 2025 May 19;31:197. doi: 10.1186/s10020-025-01235-0 (PMC12090602; doi:10.1186/s10020-025-01235-0)
Supplement: Supplementary file 1 — Supplementary Material 1 [file 10020_2025_1235_MOESM1_ESM.docx]

**Supplementary Information**

**Cytoplasmic HMGB1 promotes the activation of JAK2-STAT3 signaling and PD-L1 expression in breast cancer**

Ju-Young Han^1,2^, Woo Joong Rhee^1,3,**^, Jeon-Soo Shin^1,2,3,*^

^1^Department of Microbiology, Yonsei University College of Medicine, Seoul 03722, South Korea

^2^Brain Korea 21 FOUR Project for Medical Science, Yonsei University College of Medicine, Seoul 03722, South Korea

^3^Institute for Immunology and Immunological Diseases, Yonsei University College of Medicine, Seoul 03722, South Korea

^*^**Correspondence**: jsshin6203@yuhs.ac (J.-S.S.)

Department of Microbiology, Yonsei University College of Medicine, 50-1 Yonsei-ro Seodaemun-gu, Seoul 03722, South Korea.

Tel: +82-2-2228-1816; Fax +82-2-392-7088; Email: [jsshin6203@yuhs.ac](mailto:jsshin6203@yuhs.ac)

^**^**Correspondence**: wjrhee@yuhs.ac (W.J.R.)

Department of Microbiology, Yonsei University College of Medicine, 50-1 Yonsei-ro Seodaemun-gu, Seoul 03722, South Korea.

Tel: +82-2-2228-0794; Fax +82-2-392-7088; Email: [wjrhee@yuhs.ac](mailto:wjrhee@yuhs.ac)

**
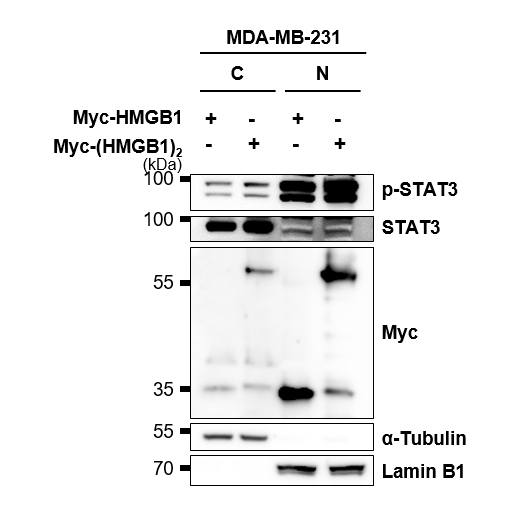
**

**Supplementary Figure S1.** **HMGB1 dimer in the cytoplasm enhances STAT3 phosphorylation in MDA-MB-231 cells.** MDA-MB-231 cells were transfected with Myc-HMGB1 and Myc-(HMGB1)_2_, followed by nuclear and cytoplasmic fractionation, and p-STAT3 expression was analyzed using Western blotting.


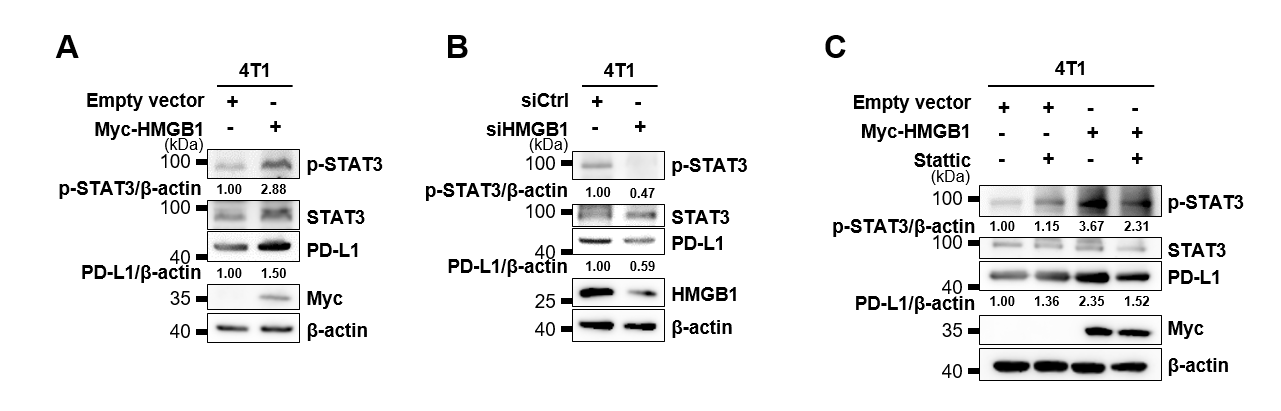


**Supplementary Figure S2. HMGB1 regulates the expression of PD-L1 through the STAT3 signaling in 4T1 cells.** **(A, B)** Immunoblots representing the expression of p-STAT3, STAT3, and PD-L1 in 4T1 cells transfected with empty vector or Myc-HMGB1 plasmid **(A)** or siCtrl or siHMGB1 **(B)**. **(C)** Immunoblots representing the expression of p-STAT3 STAT3, and PD-L1 in 4T1 cells transfected with empty vector or Myc-HMGB1 plasmid, followed by treatment with Stattic for 16 h. For all data, n = 3 samples per group.


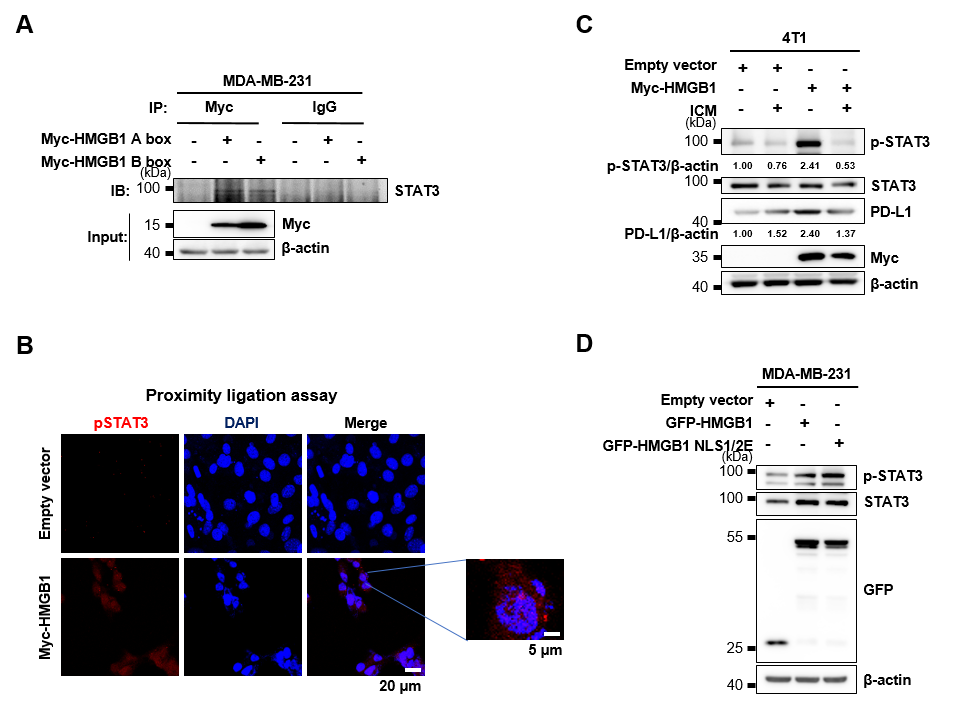
**Supplementary Figure S3. Localization of HMGB1 influences its interaction with STAT3 and promotion of phosphorylation. (A)** Myc-tagged HMGB1 A box and B box were overexpressed in MDA-MB-231 cells, and IP was performed to determine whether A box and B box bind to STAT3. **(B)** MDA-MB-231 cells were transfected with Myc-HMGB1, and the localization of p-STAT3 and HMGB1 was confirmed by PLA assay. **(C)** Immunoblots representing the expression of p-STAT3, STAT3, and PD-L1 in 4T1 cells transfected with empty vector or Myc-HMGB1 plasmid, followed by treatment with ICM for 6 h. For all data, n = 3 samples per group. **(D)** Western blot showing p-STAT3 levels upon expression of GFP-HMGB1 NLS1/2E, which contains the substitution of serine residues with glutamic acid within the NLS to mimic phosphorylation for HMGB1 cytoplasmic translocation.


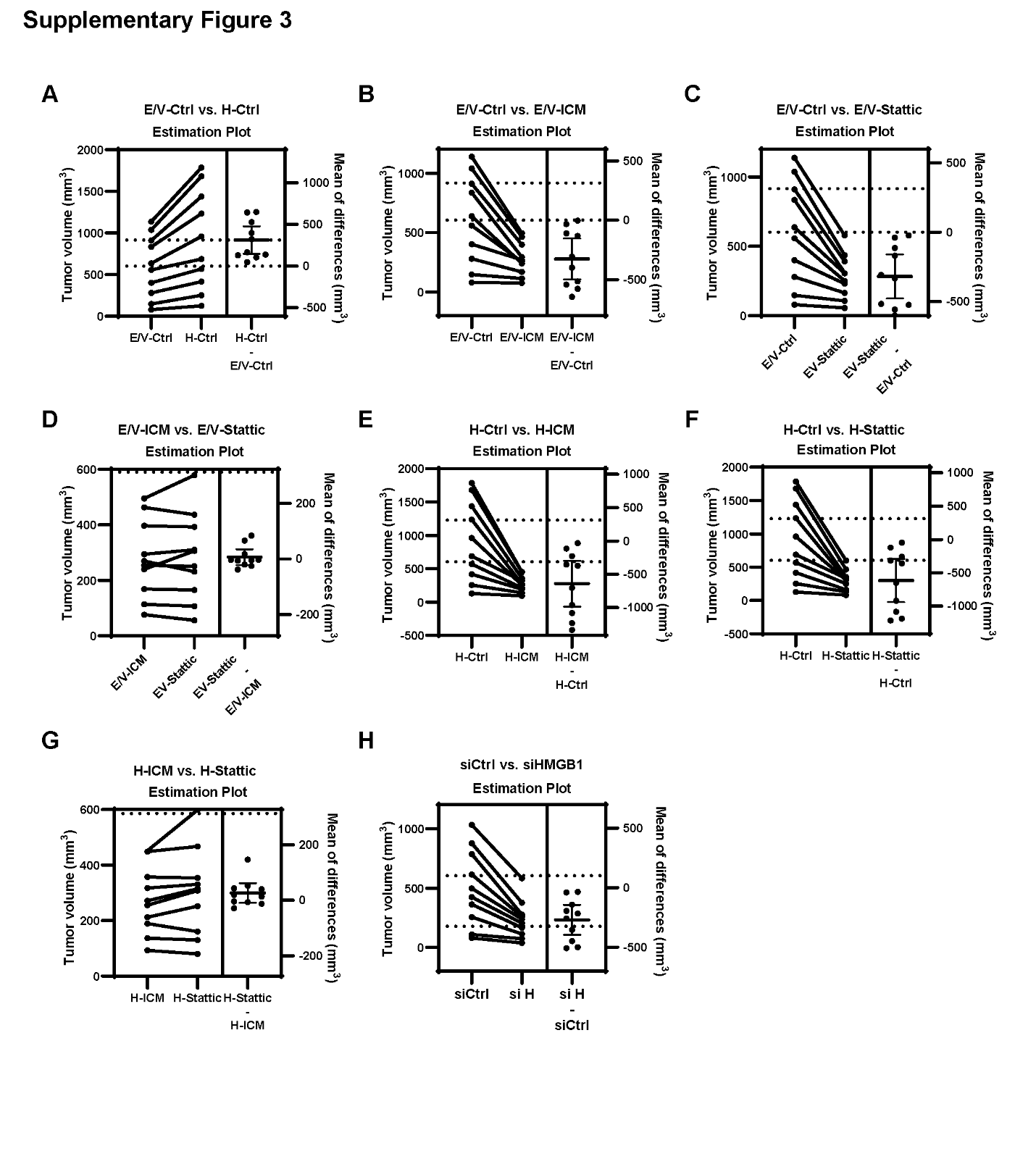


**Supplementary Figure S4.** **Estimation plots for mouse tumor volumes.** **(A–G)** Estimation plots for mouse tumor volumes between E/V-Ctrl vs. H-Ctrl **(A)**, E/V-Ctrl vs. E/V-ICM **(B)**, E/V-Ctrl vs. E/V-Stattic **(C)**, E/V-ICM vs. E/V-Stattic **(D)**, H-Ctrl vs. H-ICM **(E)**, H-Ctrl vs. H-Stattic **(F)**, and H-ICM vs. H-Stattic **(G)** groups. **(H)** Estimation plot for mouse tumor volumes between siCtrl vs. siHMGB1 groups. n = 8 mice per group for all comparisons. E/V, empty vector; H, HMGB1 overexpression.
